# Acquiring synaesthesia: insights from training studies

**DOI:** 10.3389/fnhum.2014.00109

**Published:** 2014-03-03

**Authors:** Nicolas Rothen, Beat Meier

**Affiliations:** ^1^Department of Psychology and Sackler Centre for Consciousness Science, University of SussexBrighton, UK; ^2^Department of Psychology and Center for Cognition, Learning and Memory, University of BernBern, Switzerland

**Keywords:** synaesthesia, definition, development, training, learning, acquiring, control

## Abstract

Synaesthesia denotes a condition of remarkable individual differences in experience characterized by specific additional experiences in response to normal sensory input. Synaesthesia seems to (i) run in families which suggests a genetic component, (ii) is associated with marked structural and functional neural differences, and (iii) is usually reported to exist from early childhood. Hence, synaesthesia is generally regarded as a congenital phenomenon. However, most synaesthetic experiences are triggered by cultural artifacts (e.g., letters, musical sounds). Evidence exists to suggest that synaesthetic experiences are triggered by the conceptual representation of their inducer stimuli. Cases were identified for which the specific synaesthetic associations are related to prior experiences and large scale studies show that grapheme-color associations in synaesthesia are not completely random. Hence, a learning component is inherently involved in the development of specific synaesthetic associations. Researchers have hypothesized that associative learning is the critical mechanism. Recently, it has become of scientific and public interest if synaesthetic experiences may be acquired by means of associative training procedures and whether the gains of these trainings are associated with similar cognitive benefits as genuine synaesthetic experiences. In order to shed light on these issues and inform synaesthesia researchers and the general interested public alike, we provide a comprehensive literature review on developmental aspects of synaesthesia and specific training procedures in non-synaesthetes. Under the light of a clear working definition of synaesthesia, we come to the conclusion that synaesthesia can potentially be learned by the appropriate training.

## Introduction

Synaesthesia denotes a condition of remarkable individual differences in experience characterized by specific additional experiences in response to normal sensory input. For instance, the letter A printed in black (i.e., an inducer) may elicit a red color experience (i.e., a concurrent). Already more than 100 years ago, Bleuler and Lehmann suggested that “the disposition to secondary sensations [synaesthetic experiences] is highly hereditary” (Bleuler and Lehmann, 1881, p. 49 translated from German). However, the associative nature of the phenomenon has also led researchers to ask the question whether synaesthesia or the specific associations, respectively, may be acquired through associative learning (Kelly, 1934; Howells, 1944). Despite this longstanding interest into the developmental aspects of synaesthetic experiences the exact causation is still subject to debate and it is still not clear if synaesthesia can be acquired through training inducer-concurrent associations. In this review, we first consider developmental aspects of synaesthesia and link these to the possibility that synaesthesia can be trained. Next, we review in depth the available literature explicitly concerned with the trainability of synaesthetic experiences in order to shed light on these issues.

### When to speak of trained synaesthesia?

The question whether or not synaesthesia may be acquired through training requires a clear definition of synaesthesia which can be used to assess whether any potential training procedure was successful in inducing synaesthetic experiences. Currently, there is general agreement on the following constitutive characteristics of synaesthesia. Synaesthetic experiences are *involuntarily* and *automatically* triggered by a stimulus (i.e., the inducer). Although *idiosyncratic*, synaesthetic experiences are *consistent* over time within the same individual. That is, while A may elicit a red color experience for one synaesthete, it may elicit a blue color experience for another synaesthete, but it will always elicit the same color experience for a specific individual (Grossenbacher and Lovelace, 2001; Ward and Mattingley, 2006; Ward, 2013; but see Meier et al., 2014). For most synaesthetic individuals, synaesthetic experiences have perceptual qualities that go beyond mere associations (Grossenbacher and Lovelace, 2001). For instance, grapheme-color synaesthesia entails the subjective phenomenological experience of seeing internally or externally represented colors (i.e., color photisms). Synaesthetic experiences are *unidirectional* on an explicit representational level but *bidirectional* on an implicit level. That is, in grapheme-color synaesthesia, a grapheme may elicit a color experience, but the color does not elicit the experience of the respective grapheme (Brugger et al., 2004; Cohen Kadosh and Henik, 2006; Meier and Rothen, 2007; Rothen et al., 2010; but see Cohen Kadosh et al., 2007 for the report of an exceptional case with explicit bidirectional synaesthesia). Hence, to confirm the hypothesis, that synaesthesia can be induced via training, would require the trained inducers to (i) *consistently* and (ii) *automatically* elicit (iii) the associated concurrent *experience* with perceptual qualities on a subjective phenomenological basis (iv) for the great majority of the inducers' occurrences (v) over an extended time period. Herewith, we suggest a rather conservative approach to prevent the potentially premature conclusion that synaesthesia can be acquired by means of training. Specifically, according to these criteria, know-associator synaesthetes, who do not report subjective phenomenological color experiences (as opposed to see-associator synaesthetes and projector synaesthetes), but simply know their synaesthetic associations, would not be considered as genuine synaesthetes (cf., Ward et al., 2007).

### Developmental aspects

As noted above, a genetic predisposition seems to be constitutive of synaesthesia (Baron-Cohen et al., 1996; Ward and Simner, 2005; Tomson et al., 2011). That is, synaesthesia tends to run in families (Asher et al., 2009), although individual members of a family may experience different forms of synaesthesia (Barnett et al., 2008). For instance, while one family member may experience colors for letters and numbers (i.e., grapheme-color synaesthesia), another family member may experience spatial arrangements in response to sequence based concepts such as the days of the week (i.e., sequence-space synaesthesia). However, even when synaesthesia occurs within a family, typically not all members are concerned. The presence of one form of synaesthesia in an individual tends to increase the likelihood that the same individual also experiences another form of synaesthesia (Sagiv et al., 2006b). On a neural basis, synaesthesia is associated with functional and structural changes. Grapheme-color synaesthesia, which is currently the best studied form of synaesthesia, is associated with increased structural connectivity in occipito-temporal and parietal regions (Rouw and Scholte, 2007; Rouw et al., 2011; Banissy et al., 2012; Specht, 2012). The structural changes seem to be associated with functional changes during the perception of synaesthetic color experiences (Hubbard et al., 2005; Weiss et al., 2005; but see, Hupé et al., 2012). Increased activation in hV4 (i.e., involved in human color perception) is hypothesized to reflect the perception of synaesthetic color experiences (Ramachandran and Hubbard, 2001a,b). Increased activation in parietal regions around the sulcus intraparietalis and gyrus angularis are thought to reflect binding processes between the synaesthetic inducer and the synaesthetic concurrent experience (Esterman et al., 2006; Muggleton et al., 2007; Rothen et al., 2010). Moreover, some researchers suggested that synaesthetic experiences have real perceptual qualities which may lead to a performance advantage in perceptual tasks (Ramachandran and Hubbard, 2001a; Hubbard et al., 2005; Sagiv et al., 2006a; Ward et al., 2010; but see, Mattingley et al., 2001; Edquist et al., 2006; Rothen and Meier, 2009). Others have argued that synaesthetic experiences are represented as nodes in a semantic network and that synaesthesia is not a pure perceptual condition (Meier, in press). Synaesthesia is generally regarded as a congenital condition and, as such, sometimes also termed “developmental synaesthesia” to distinguish from “metaphorical synaesthesia” (e.g., screaming colors).

However, a genetic predisposition does not necessarily mean that the respective condition will indeed develop. Even in the case where a genetic predisposition for synaesthesia eventually results in synaesthetic experiences, it is rather unlikely that the specific synaesthetic associations are determined by genes. This is especially the case because most synaesthetic experiences are triggered by cultural artifacts, as for instance in grapheme-color synaesthesia (Rich et al., 2005; Simner et al., 2009), sequence-space synaesthesia (Eagleman, 2009), and lexical-gustatory synaesthesia (i.e., words elicit taste experiences; Ward and Simner, 2003). Crucially, synaesthetic consistency is only achieved after conceptual knowledge about the synaesthetic inducer has been acquired (Simner et al., 2009). In line with this notion, it has been suggested that it is the conceptual representation of the synaesthetic inducer which triggers the specific concurrent experience (Nikolić et al., 2011; Rothen et al., 2013), or at least that the conceptual representation of a synaesthetic inducer is sufficient to elicit the concurrent experience (Dixon et al., 2000, 2006). Moreover, there are cases for which the specific synaesthetic associations are related to prior experiences (for example colored alphabet toys; Witthoft and Winawer, 2006, 2013). Similarly, large scale studies show that grapheme-color associations in synaesthesia are not completely random (Rich et al., 2005; Simner et al., 2005). However, these studies failed to find correlations with colors for letters and numbers in children's books (Rich et al., 2005). Crucially, also the structural neural basis of synaesthesia may be a consequence rather than a precondition for synaesthetic experiences. In line with this notion, synaesthetic experiences can be induced post-hypnotically in non-synaesthetic individuals who do not possess the structural neural basis of synaesthesia. Hence, synaesthetic experiences may be a result of functional disinhibition between relevant brain areas, but structural changes are not necessary for conscious synaesthetic experiences (Cohen Kadosh et al., 2009). Similarly, developmental synaesthetes can acquire synaesthetic experiences for novel inducers within minutes (Mroczko et al., 2009). Furthermore, the early onset of synaesthesia does not categorically exclude the possibility that synaesthetic experiences can be acquired later in life. For instance, there is evidence for a higher prevalence of synaesthesia among artists and meditators which suggests that synaesthesia may be cultivated (e.g., Walsh, 2005; Rothen and Meier, 2010b).

To summarize, a learning component is inherently involved in the development of specific synaesthetic associations, in particular when the inducers are cultural artifacts (as in grapheme-color synaesthesia). Some researchers have hypothesized that associative learning is the critical learning mechanism, where the recall event consists of implicit imagery of the synaesthetic concurrent (Albright, 2012). Hence, it seems plausible that synaesthetic experiences may be acquired, and there is already clear evidence that at least some aspects of synaesthesia can be acquired through training. Training studies may provide further insights into the mechanisms which are at play during the development of synaesthesia.

### Two research trends of public interest

**Figure 1:**
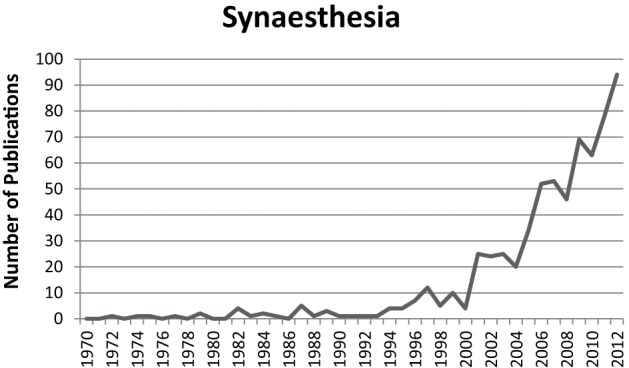
**Number of publications per year in the field of synaesthesia research**. The figure represents the hits for the search terms “synaesthesia OR synaesthesia” in the title, abstract, or keywords. Source: Scopus (17/09/2013).

Over the last 10–20 years there was an increase in scientific publications about synaesthesia (Figure 1). Earlier publications were mainly concerned with documenting specific types of synaesthesia and demonstrating their genuineness (Baron-Cohen et al., 1987). Besides this, later publications were also concerned with documenting the characteristics associated with synaesthesia. There is empirical evidence suggesting that synaesthesia is associated with a specific profile of enhanced memory performance (Yaro and Ward, 2007; Rothen and Meier, 2010a; Radvansky et al., 2011; Rothen et al., 2012; Meier and Rothen, 2013b), increased creativity (Rich et al., 2005; Ward et al., 2008; Rothen and Meier, 2010b), and increased self-rated imagery (e.g., Barnett and Newell, 2008; Meier and Rothen, 2013a). The increase in scientific publications led to documentary films (e.g., BBC) and articles in popular science magazines (e.g., Ramachandran and Hubbard, 2003; Lehrer, 2007; Bouska, 2013). Consequently, public awareness and interest in this—to the public—seemingly peculiar condition was rising. Possibly due to the positive effects associated with synaesthesia, it became a question of more general interest to what extent synaesthesia can be learned or trained. This is reflected on websites and blogs on the internet which discuss the issue and relevant findings from research studies. Moreover, a “market” exists to promote synaesthesia related products. For example, synaesthetic training courses are offered to improve creativity and memory (Samarajiwa, 2014; Söffing, 2014; cf. also, Preiser, 2011). Synaesthesia training has also been suggested as a form of psychotherapy (Synästhetische Gedanken [Synaesthetic Thoughts], 2014; cf. also, Lewis, 2012). Interestingly, even synaesthetes seem to ask how they can enhance their experiences, for example in order to increase their creative artistic output (Kann ich meine Synesthäsie weitertrainieren, damit ich sie nutzen kann? [Is it possible to train my synaesthesia for further benefits?], 2014). However, there is no scientific basis for the claims that synaesthesia training can actually provide for the cognitive profile associated with synaesthesia and its advantages (and potential disadvantages). Therefore, the question, whether synaesthesia can actually be acquired via training, is important for synaesthesia researchers and to inform the public.

**Figure 2:**
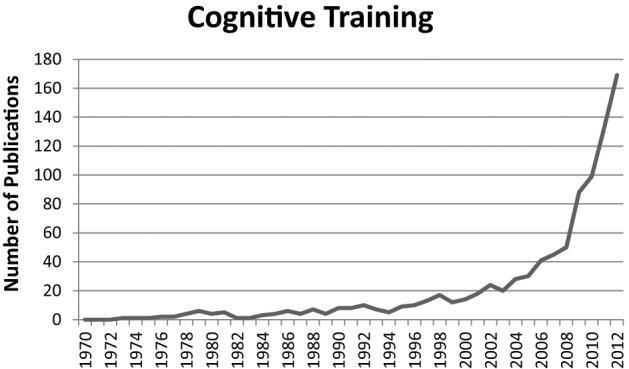
**Number of publications per year in the scientific field of cognitive training**. The figure represents the hits for the search term “cognitive training” in the title, abstract, or keywords. Source: Scopus (17/09/2013).

Independent from synaesthesia research, cognitive training has also gained a lot of interest over the last two decades (Figure 2). The correspondent scientific publications even outnumber those about synaesthesia. Cognitive training studies are mainly concerned with transfer effects. That is, whether training one cognitive process affects other cognitive processes that were not trained. For instance, recent findings suggest that working memory training can also enhance performance in intelligence tests (e.g., Jaeggi et al., 2008, 2011). Accordingly, there is great public interest in cognitive training, resulting in similar effects as the interest in synaesthesia. That is, there are numerous blogs and internet pages on cognitive training, as well as articles in popular science magazines (e.g., Sinha, 2005; Robertson, 2010). Moreover, cognitive training programmes are sold in form of computer games (e.g., Nintendo's “Dr. Kawashima's Brain Training: How Old is Your Brain?”).

From this point of view, synaesthesia training studies are important to understand the plasticity of the cognitive system more generally. As there is evidence that synaesthesia is associated with cognitive benefits, and cognitive training can lead to transfer effects, it is a promising avenue for future research to investigate transfer effects of synaesthesia training.

## Acquiring synaesthesia: state of research

### Training studies

So far, seven synaesthesia training studies have been published. All were explicitly concerned with the question whether *consistent* and *automatic* concurrent *experiences* with perceptual qualities on a subjective phenomenological basis can be induced. These studies will be reviewed in depth. They are thematically and roughly chronologically ordered (see Table 1 for a summary).

**Table 1:** **Summary table of training procedures and effects**.

**Study**	**Type of synaesthesia**	***N* participants**	**Training**	***N* trials**	***N* congruent trials**	***N* incongruent trials**	**Ratio (congruent/incongruent)**	**Duration**	**Effect**	**Synaesthetic experience**
Kelly, 1934	Sound-color	18/18	Associative learning	Min 280	All	NA	1:0	Several weeks	NA	No
		6/18		Min 2000	All					No
Howells, 1944	Sound-color	8	Associative learning	ca. 30,000	95%	5%	19:1	Several weeks	Stroop type interference, overadjustment toward opponent color	Yes
Meier and Rothen, 2009	Letter-color	20	Associative learning	3360	1680	1680	1:1	7 days	Stroop interference, but no synaesthetic conditioning	No
Rothen et al., 2011	Digit-color	20/40	Associative learning	4800	2400	2400	1:1	10 days	Stroop type interference in 1 of 2 tasks	No
		20/40	Associative learning and mental imagery	2480	2480	NA	1:0	10 days	Stroop type interference in 2 of 2 tasks	No
Rothen et al., 2013	Swimming-style color	1	Associative learning	9600	4800	4800	1:1	20 days	Stroop type interference, but no synaesthetic conditioning	No
Colizoli et al., 2012	Letter-color	15	Incidental learning (reading)	Mean of 105,660 words	All	NA	1:0	2–4 weeks	Stroop interference, but no perceptual crowding	No
Kusnir and Thut, 2012	Letter-color	28 (Exp. 1)	Incidental learning (visual search)	1620 (of interest 1080)	900	180	5:1	2 days	Facilitated target detection for congruent targets, no Stroop effect	No
	Letter-color	22 (Exp. 2)	Incidental learning (visual search)	2430 (of interest 1620)	1352	268	5:1	3 days	Facilitated target detection for congruent targets, color opponency effect	No
Cohen Kadosh et al., 2005	Digit-color	6	Associative learning	8100	900	7200	1:8	5 days	Colors did not implicitly activate numerical magnitude	?
Nunn et al., 2002	Word-color	10	Associative learning	To criterion	All	NA	1:0	Few minutes	Less activation in V4/V8 compared to synaesthetes	?
Brang et al., 2011	Grapheme-color	24	Guess-and-check	To criterion	NA	NA	NA	ca. 15 min	Contextual priming based on cognitive rather than perceptual processes	?
Niccolai et al., 2012	Grapheme-color	7	Training battery (several tasks)	?				6 days	Stroop interference, based on cognitive rather than perceptual processes	?

#### Early attempts: sound-color synaesthesia via conditioning

In a first study, it was tested whether sound-color synaesthesia can be induced artificially by means of associative learning/conditioning (Kelly, 1934). The following introspective criterion was applied to test the success of the study: “If the tones became capable of arousing spontaneous sensations or images of color in the subjects, then and only then, could it be said that a true conditioned response had been established.” (Kelly, 1934). Eight different accordion tones of a complete octave and seven different projected color squares were used to create tone-color pairs (C and C1 were both white). Eighteen participants, all non-synaesthetes but one sequence-space synaesthete, attended a passive training procedure including multiple sessions during which the pairs were presented (tone and color simultaneously) for a duration of ~9 s. The interval between two pairs was 1.5 s, and the scale was always presented in ascending and then descending order several times per experimental session. Over the duration of the experiment, six participants were presented with at least 2000 repetitions per stimulus pair. For the remaining participants, the minimum was 280 repetitions per pair. Once a week, participants were presented with 40 tones alone (each tone five times) and were asked to indicate the color associated with the tone, and whether they had a sensation of color for the tone. None of the participants reported any color sensations. The results showed no evidence at all that synaesthesia may be induced artificially. Interestingly, Kelly (1934) pointed out that “It has been argued by some that a purely physiological theory is sufficient without including the assumption that conditioning occurs. A crucial test of whether conditioning does supplement a physiological factor in the production of chromaesthesia [synaesthesia] could be made by attempting to destroy and change the color-tone associations in bona fide cases of colored hearing [sound-color synaesthesia]. If it could be shown that it is possible to experimentally destroy the linkages reported, it would offer almost incontrovertible evidence that conditioning had played a role in the production of synaesthesia.”

Ten years later, another study was conducted with the aim to establish sound-color synaesthesia in a group of eight participants (Howells, 1944). Using a conditioning paradigm, two tones (middle C and G above) and two colors (red and green) served as stimuli. Participants were required to keep their eyes closed until the onset of the tone, to open their eyes, and to report the color which had its onset immediately after the tone. The tone was presented for the duration of 2 s, and its offset was simultaneous with that of the color. Next, participants were required to close their eyes again for the next trial. Middle C was paired with red and G was paired with green for 95% of the trials. The pairings were reversed for the remaining 5% of the trials. These stimulus pairs were presented in different predetermined random orders. Participants were informed about the correctness of the response after every trial. After 5000 trials of presenting the colors at maximum saturation, the hue was reduced for 50% of the trials (including 50% of the reversed tone-color pairings). As dependent variable, error rates were assessed for reversed tone-color pairings in sets of 50 trials. After the first 5000 trials for which almost no errors occurred, the error rates increased almost linearly to ~17 over the course of the study. Each participant was presented with ~30,000 trials in total. The explanation, offered by the participants for the observed effect, was that “The habitual set, or expectancy of seeing a given hue after hearing the paired tone, became so strong that it overpowered the conflicting perceptual influence of the hue actually supplied, with the result that pale green was actually seen as pale red, or vice versa” (Howells, 1944, p. 96). In order to determine whether the participants indeed experienced colors two of the participants were also tested with additional behavioral tasks (100 trials per participant and task). During one task, the participants were presented with the same stimuli as during the conditioning paradigm. However, instead of pale colors the participants were presented with a white stimulus in 50% of the cases, the other 50% of the cases consisted of saturated colors. One participant suspected that the “pale” stimuli were actually white, but reported that they still appeared in color to him. The other participant did not realize that the stimuli were white. During the other task, the participants were again presented with the original tones and the original colors. Tones and colors were presented simultaneously, one pair at a time, in random order. The two colors were presented by means of two light beams which could also be mixed via the adjustment of a slide. The task was to adjust the presented color to appear white. Suggesting an associated color experience (i.e., perceptual effect), it was found that for the lower (red) tone an over-adjustment toward green occurred, and for the higher (green) tone an over-adjustment toward red occurred. Howells (1944) concluded that cumulative conditioning had led to synaesthetic experiences.

#### Grapheme-color synaesthesia via associative learning

It was not until more than half a century later before the next training study was conducted. The study was realized to investigate whether synaesthetic Stroop effects are a valid diagnostic criterion for synaesthesia, and whether psychophysiological consequences of grapheme-color synaesthesia can be established by training specific letter-color associations (Meier and Rothen, 2009). Twenty non-synaesthetes were trained to learn four different letter-color associations; A—red, B—green, C—yellow, and D—blue. Participants had to press one of two designated keys as quickly and accurately as possible if, at the center of the computer screen, a letter was presented in its correct color, and the other of two designated keys if a letter was presented in its incorrect color. Each session consisted of 480 trials. Each letter was presented 60 times in its correct color and 20 times in each of the other three incorrect colors in random order. Feedback about mean reaction time and proportion correct was given after every session to enhance motivation. There was one training session per day on 7 consecutive days. Every participant was presented with a total of 3360 trials, half of which were correct letter-color pairings. Mean accuracy over all training sessions was 98%.

**Figure 3:**
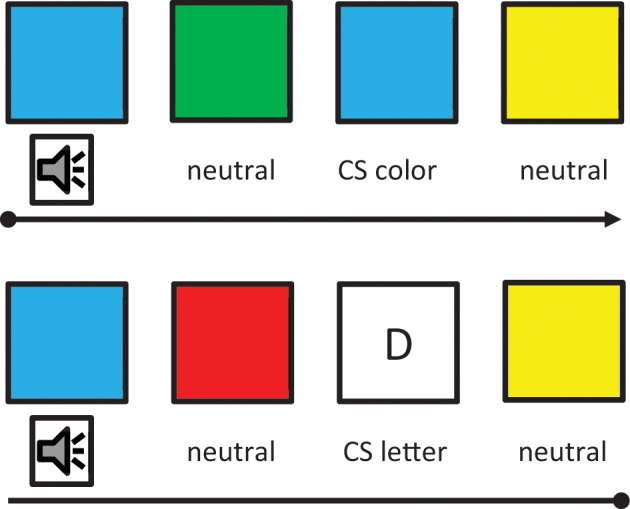
**Synaesthetic conditioning**. In this particular example, the letter D was associated with blue during the training. That is, blue squares not followed by the startling sound acted as CS color and the letters as CS letter. The remaining colors were neutral. Each square represents one trial with the first trial in the upper left corner and the last trial in the lower right corner (moving from left to right). Adopted from Meier and Rothen (2009).

**Figure 4:**
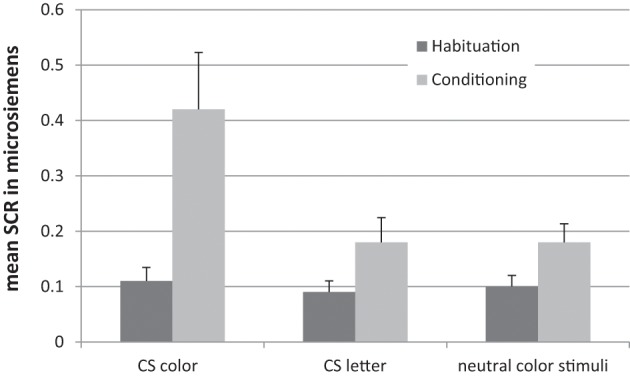
**Results from the synaesthetic conditioning paradigm showing a conditioning response for the CS color, but not the CS letter and neutral color stimuli for trained non-synaesthetes**. Error bars represent standard errors. Adopted from Meier and Rothen (2009).

After the training, participants were tested with a synaesthetic Stroop task and a synaesthetic conditioning task (Meier and Rothen, 2007). During the Stroop task, participants were presented with two letters. For half of the trials, the letters were presented in their congruent colors (i.e., according the trained association). For the other half of the trials, the letters were presented in an incongruent color (i.e., color associated with the other letter). A significant Stroop effect was found for the 20 trained participants. This was not the case for 20 untrained non-synaesthetic controls. During the synaesthetic conditioning task participants were required to attend to colored squares. One of the squares was white and contained a letter associated with the color of another square by means of the training. This specific color was followed several times by a loud sound to provoke a conditioned startle reaction to that color (i.e., CS color). Neither the letter (CS letter) nor the other colors (neutral) were followed by the startling sound (Figure 3). Conditioning was measured by means of event-related skin conductance responses (SCRs), with higher SCRs indicating higher autonomic arousal. For CS color trials (but not for CS letter trials and neutral filler trials), SCRs were significantly increased during conditioning in comparison to a preceding habituation phase without the startling sound (Figure 4). That is, in contrast to genuine synaesthetes (Meier and Rothen, 2007; Rothen et al., 2010), non-synaesthetes, trained on letter-color associations, did not show a significant increase for the CS letter during conditioning. Moreover, none of the participants reported having color experiences for the trained letters. To summarize, the training was successful in creating automatic letter-color associations, as measured by the Stroop task, but not in creating synaesthetic experiences, as indicated by the absence of a synaesthetic conditioning effect and the subjective phenomenological reports. Thus, this cannot be regarded as synaesthesia and hence, synaesthetic Stroop effects are not a valid diagnostic criterion for synaesthesia.

#### Grapheme-color synaesthesia via adaptive/non-adaptive training

**Figure 5:**
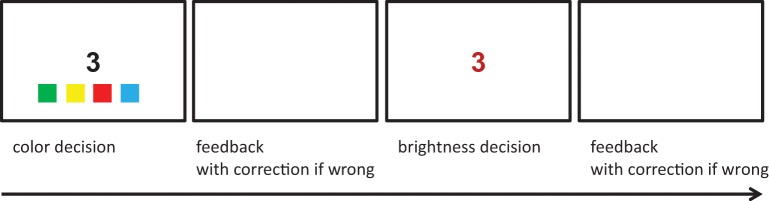
**Adaptive training**. Exemplary depiction of a trial. Adopted from Rothen et al. (2011).

In a follow-up study, the same training (i.e., non-adaptive) was compared with an adaptive training procedure (Rothen et al., 2011) to test for associative learning of automatic number-color associations, as they exist in grapheme-color synaesthesia. Forty non-synaesthetic participants were trained with either the non-adaptive or the adaptive version of the training on 10 consecutive days (*N* = 20 per training procedure). They were instructed to learn the following associations: 3—red, 4—green, 5—yellow, and 6—blue. Apart from these different grapheme-color associations, the non-adaptive training paradigm was identical to the previously introduced training (Meier and Rothen, 2009). That is, in the non-adaptive training, participants were presented with a total of 2400 match and 2400 non-match trials over the duration of the training. In the adaptive training, participants had to indicate as quickly and accurately as possible, by pressing one of four distinct keys, which color was associated with a black digit, presented centrally at the computer monitor. The color-key mapping changed on a trial-to-trial basis. Participants were provided with feedback after every trial and presented with a square in the correct color if their response was wrong. Next, they were presented with the digit in its correct hue but wrong brightness and had to indicate if the presented color was lighter or darker than the correct color. The brightness manipulation followed a staircase procedure depending on the previous response of a given color. Participants received also feedback for this response, and the digit was presented in its correct color if their response was wrong (Figure 5). Each of the daily sessions consisted of 248 trials. Hence, participants were presented with a total of 2480 trials over the duration of the training. For both versions of the training, mean accuracy was 96%.

Before and after the training, participants were tested with two synaesthetic priming tasks (cf., Gebuis et al., 2009). In one version of the task, participants were required to indicate a target color by pressing one of four specified keys. The target color was preceded by digits to which the associated color was either congruent or incongruent to the target. In the other version of the task, participants were required to indicate a target digit by pressing one of four specified keys. In this version, the colors were used as primes. After the training, the results revealed priming effects for both versions of the task in the adaptive training group. In the non-adaptive training group, priming was restricted to the digit-decision task. On average, the adaptive training group showed larger priming effects. However, none of the participants reported synaesthetic experiences after the training. Hence, adaptive training seems to be generally more effective in creating automatic digit-color associations than non-adaptive training, but it seems not sufficient enough in order to create synaesthetic experiences when applied for a short duration of 10 consecutive days.

#### Swimming-style color synaesthesia via associative learning

Using the non-adaptive training with pictograms of swimming-styles on 20 consecutive days (i.e., 4800 matching and 4800 non-matching trials), Rothen et al. (2013) tested whether swimming-style color synaesthesia can be acquired. According to the associations of a genuine swimming-style color synaesthete, a non-synaesthete learned the following associations: butterfly—red, breaststroke—blue, backstroke—white, and crawl—pale yellow. As it was the case for the synaesthete, the trained control showed priming effects in a color decision task when primed with pictograms of swimming styles and also in a swimming-style decision task when primed with colors (cf., Rothen et al., 2011). However, only the synaesthete showed a generalized conditioned response for pictograms of a swimming-style associated with the color that was coupled with a loud startling sound (i.e., synaesthetic conditioning; Meier and Rothen, 2007, 2009; Rothen et al., 2010). Crucially, the trained control did not report phenomenological color *experiences* in association with swimming-styles.

#### Grapheme-color synaesthesia via reading books with colored letters

In a related study, Colizoli et al. (2012) asked 15 non-synaesthetic controls to read books with colored letters. That is, each participant learnt a distinct set of associations between the letters a, e, s, t and the colors red, orange, green, blue. Moreover, participants were asked to use a web applet that colored the letters on internet pages. They were instructed to use it whenever they were reading for a significant amount of time. On average, participants read 105,660 words over the course of 2–4 weeks. A Stroop task was conducted before and after the reading-training as in the study of Meier and Rothen (2009). After the reading-training, but not before, participants showed a significant Stroop effect. The effect was stronger in its magnitude for lower case letters, which appeared more often during the course of the training than upper case letters. Moreover, a near significant correlation was found between the rating of the statement “I am experiencing color when thinking about certain letters” and the magnitude of the Stroop effect. However, this was not the case for the statement “I am experiencing color when I see certain letters.” The authors also tested whether the learned associations would enhance performance in a perceptual crowding task. The task was adapted from a study in which a performance advantage was found for genuine grapheme-color synaesthetes over non-synaesthetic controls (Hubbard et al., 2005).

During a trial of this task, a unique black target letter surrounded by four identical black flanking letters was presented for a brief duration, randomly, either on the left or right side of a fixation-cross at the center of a computer screen. Participants were required to identify the target letter. There were two conditions, a set of trials for which the trained letters served as target letters, with one of the other trained letters as flankers, and a baseline condition with the letters d, f, g, and o. To test for inherent differences between the letters of the two conditions, a non-trained group of controls (*N* = 30) was tested with the same task. However, no performance advantage was found for the trained letter condition in the training group in comparison to the baseline condition and the untrained control group. Participants were asked in a surprise retest 6 months after the training to report the colors when given the letters. On average they were 98% correct at identifying the colors overall and 40% correct at actually remembering the specific associations. Similarly to previous training studies, the findings suggest that the training was successful in inducing automatic letter-color associations, but not in inducing the perceptual aspects of synaesthesia (i.e., synaesthetic *experience*).

#### Grapheme-color synaesthesia via incidental associative learning

**Figure 6:**
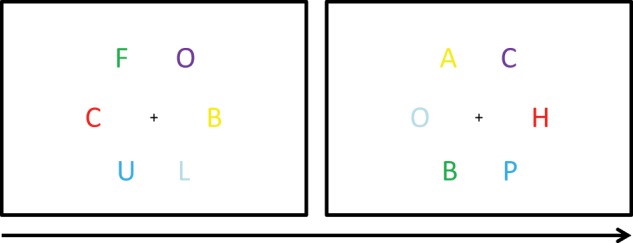
**Visual search task**. Two exemplary trials. The two target letters of interest (i.e., H and U) appeared more often in a specific associated color. The other letters appeared in each color equally often. Adopted from Kusnir and Thut (2012).

In another study, Kusnir and Thut (2012) tested to what extent synaesthesia-like letter-color associations may be implicitly learned by non-synaesthetes. A visual search task with trials containing circular arrays of six colored letters around a fixation cross at the center of the computer monitor was used as a training paradigm (Figure 6). Participants were instructed to indicate by keypress whether the target letter (one of three pre-specified target letters, i.e., H, U, and S) was presented left or right of the fixation cross. The distractor letters were A, B, C, F, L, O, and P. Target and distractors were selected randomly on each trial. Each of the letters was presented in a different color (red, blue, cyan, yellow, green, and magenta). Learning of letter-color associations was established to occur incidentally via manipulating the frequency at which letters occurred in a specific color. That is, two of the target letters (i.e., H and U) were biased to appear 5 out of 6 times in a respective target color and 1 out of 6 times in any other color. The likelihood of the two color-biased target letters together, to be observed in their respective biased color (i.e., congruent), was 55.6%. The likelihood of the two target letters together to be observed in the respective color of the other color-biased target (i.e., incongruent) was 2.2%. The remaining target letter was not color-biased and appeared in every color with equal likelihood (5.6%). However, when considered on their own, any of the target letters or target colors occurred with equally high likelihood across trials. In a first experiment, 28 non-synaesthetes were tested. To manipulate the depth of color processing, 14 participants were informed about the letter-color manipulation and 14 were kept naïve. The training consisted of a total of 1620 trials and was conducted on 2 consecutive days. In a second experiment, 22 non-synaesthetes participated and all were informed about the likelihood of the letter-color pairings. Moreover, for half the participants the two target colors of interest were opponent colors, but not for the remaining half of the participants. Notably, greater interference for opponent colors, in comparison to non-opponent colors, was reported as evidence for early stages in the visual processing of genuine synaesthetic colors (Nikolić et al., 2007). The training consisted of a total of 2430 trials, and the task was performed over three sessions within 1 working week.

A significant difference in search performance between congruent and incongruent letter-color pairings was found in both experiments. That is, congruent letter-color pairings facilitated target detection. Incongruent letter-color pairings impaired target detection. Crucially, color information *per se* would not have enhanced target detection in the congruent, relative to the incongruent, condition but equally across these conditions. Greater interference for incongruent targets was found in the opponent color condition than the non-opponent color condition in Experiment 2. After each of the two training sessions, participants of Experiment 1 were tested with two additional Stroop paradigms similar to those used in previous studies (Meier and Rothen, 2009; Colizoli et al., 2012). One version was conducted with saturated colors, the other version was conducted with unsaturated colors to account for the possibility of weak acquired synaesthetic color experiences. Generally, on a group level no Stroop effects were found. Further explorations revealed a Stroop effect in the deep color processing group for the faint version of the task. Crucially, however, none of the trained participants reported color *experiences* for (the trained) letters at the end of the experiments. Because of this and the weak evidence for Stroop effects in the faint version of the task, the authors concluded that the learned associations were qualitatively different to synaesthesia.

#### Related studies

The following four studies are important to the aim of this review because non-synaesthetes, trained on grapheme-color associations, were included. However, the participants were not explicitly asked about their phenomenological experiences. That is, the trained associations were regarded as purely semantic. The studies shall be summarized each very briefly in one paragraph. An overview and additional information can be found in Table 1.

**Figure 7:**
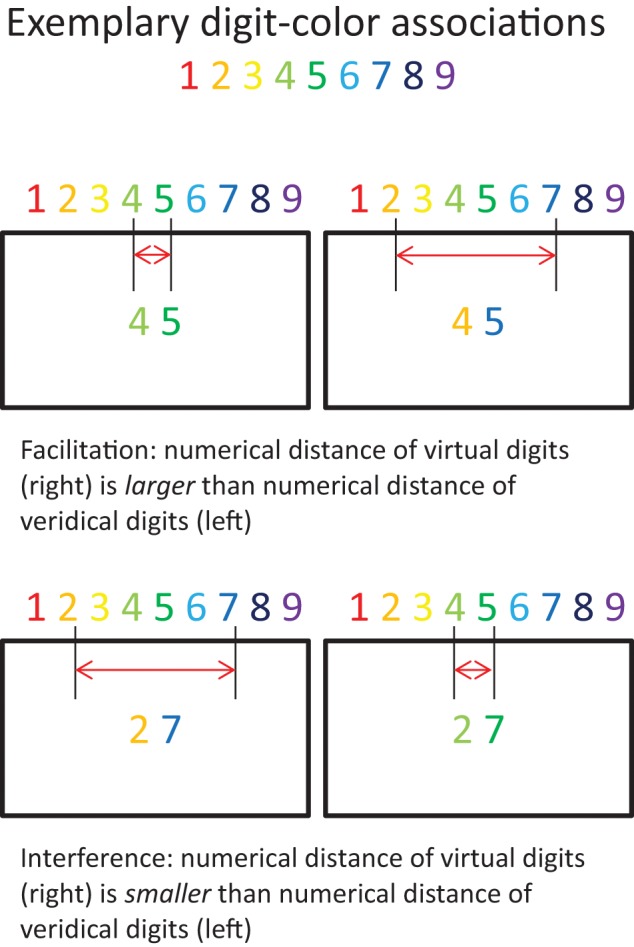
**Implicit numerical magnitudes**. Exemplary depiction of experimental conditions: Facilitation and interference effects due to activation of implicit numerical magnitudes via color information. The red horizontal arrows represent the virtual numerical magnitude as indicated by the colors. Adopted from Cohen Kadosh et al. (2005).

Cohen Kadosh et al. (2005) tested whether training digit-color associations in non-synaesthetes (*N* = 6) can result in implicit numerical magnitude representation for colors. At the beginning of the session, participants were presented with the specific associations. In the next task, they had to indicate as quickly and accurately as possible if the background color of the computer screen matched the color associated with a centrally presented gray digit (1–9) by pressing one of two distinct keys. Incorrect responses were followed by auditory feedback. Each digit-color combination was presented equally often. That is, the ratio between matching and non-matching conditions was 1:8. The task consisted of 20 blocks, including 81 trials each. This procedure was repeated on 5 consecutive days. The training consisted of a total of 8100 trials. Moreover, the trained participants and two grapheme-color synaesthetes completed a color congruity task (Figure 7), during which they had to decide which of two colored digits represented the larger magnitude. Only the two synaesthetes showed the anticipated facilitation effect. That is, they responded faster when the actual colors (of the digits) on the screen represented a larger numerical distance in comparison to a smaller numerical distance (Figure 7; upper right compared to upper left). No interference effects were found (Figure 7; lower right compared to lower left). The results suggest that only genuine synaesthesia provides for implicit bidirectional representations.

One of the first studies designed to test for the perceptual reality of synaesthetic color experiences included a control group of ten non-synaesthetic participants with trained word-color associations (Nunn et al., 2002). The stimulus material consisted of eight different word-color associations that were similar to those of the synaesthetes. The learning procedure consisted of a 2 × 4 grid with the different colors presented on a computer monitor. Upon clicking on a color, the corresponding word was presented via headphones, the computer monitor filled with the color and remained on the monitor until another color was selected. Learning was tested by the presentation of a word via headphones for which the corresponding color had to be selected. Word presentation during testing was random. No feedback was given. Learning and testing cycles were continued until the criterion of 100% accuracy on five consecutive trials was reached. In contrast to synaesthetic experiences of word-color synaesthetes, who underwent functional Magnetic Resonance Imaging (fMRI) in the same study, imagined colors in trained controls did not activate color selective brain regions (i.e., V4 and V8). The findings suggest that the training did not lead to color associations of similar perceptual quality as synaesthetic experiences.

In order to assess conceptual and perceptual aspects of synaesthetic experiences, Brang et al. (2011) conducted an electroencephalogram (EEG) study with genuine grapheme-color synaesthetes (*N* = 12) and trained controls (*N* = 24). During the training, achromatic graphemes were presented at the center of the screen, surrounded by ten distinct colored blocks (i.e., red, orange, yellow, green, blue, purple, black, brown, white, and pink) whose position was randomized on each trial. By clicking on the colors, participants had to learn ten grapheme-color pairings in a guess-and-check fashion. Participants were trained, until they were able to correctly identify the color of each of the ten graphemes two times in a row without any errors. The duration of the training was ~15 min on average. The synaesthetes, the trained controls, and an additional non-trained control group (*N* = 12) were tested with a contextual priming paradigm. That is, participants were presented with sentences like “The Coca-Cola logo is white and X.” On a given trial, “X” was either a color patch, color word, or a grapheme, which was either congruent or incongruent with the representation of “X” (i.e., red in our example). That is, the presented graphemes were associated to either congruent or incongruent colors (relative to the semantic context) for synaesthetes and trained controls. For the non-trained control groups, the graphemes were presented in actual colors. For all groups, half of the sentences of each type ended with a congruent stimulus and half with an incongruent stimulus. Event-related potentials (ERPs) were measured for the representation of “X.” Representing perceptual processes, early ERPs to graphemes were significantly affected by congruency in the synaesthete group only but not in the control groups (unless they were presented with colored graphemes). Representing semantic processes, late ERPs to graphemes were significantly affected by congruency in all groups. The groups were not differentially affected by congruency in the other conditions. Thus, the findings suggest that the training procedure in this study was not sufficient to induce synaesthetic color *experiences* to graphemes beyond the level of semantic associations.

The main goal of a related EEG study (Niccolai et al., 2012) was to compare the neural processes associated with synaesthetic priming (similar to previous studies; e.g., Rothen et al., 2011) for congruent and incongruent trials between seven genuine grapheme-color synaesthetes and seven trained controls. The controls were required to learn specific grapheme-color associations (letters and digits; average *N* = 30) with a computer based training at home on 6 consecutive days. The training consisted of (1) selecting the color patch corresponding to a grapheme in a three-alternative forced choice paradigm, (2) assigning “correctly” four different color-patches to four different graphemes, (3) reporting the grapheme associated with a presented color patch. The training was not further specified. It lasted 18 min on average. The error rate declined significantly from start to end with an average rate of 4.3%. Synaesthetes and trained controls showed similar synaesthetic priming effects (i.e., faster responses to congruent than incongruent trials). In line with Brang et al. (2011), congruency was more likely to affect early ERP components in the synaesthete group and late components in the trained control group. Hence, the results suggest that controls did not *experience* synaesthetic colors after the training.

## Discussion

To summarize, none of the studies outlined above provided direct evidence that synaesthesia was induced by means of the specific training procedure (see Deroy and Spence, 2013 for similar conclusions). Nevertheless, the findings are promising and clearly demonstrate that certain aspects of synaesthesia can be mimicked in non-synaesthetes. Especially the differences in subjective phenomenological reports between synaesthetes and trained non-synaesthetes and the respectively associated cognitive, behavioral, and neural profile may provide further insights into cognitive science.

The most compelling evidence that synaesthesia can be acquired later in life by means of training comes from one of the earliest studies. As described by the author, the explanation offered by the participants indeed points to *consistently* and *automatically* elicited concurrent *experiences* with perceptual qualities on a subjective phenomenological basis (Howells, 1944). Also more recent studies provide hints that synaesthesia can be acquired by means of training. For example, the finding of greater interference for incongruent targets in the opponent color condition than the non-opponent color condition may reflect a perceptual effect as documented in developmental synaesthesia (Kusnir and Thut, 2012 Experiment 2). However, an alternative interpretation, namely that opponent colors may be more easily conceptualized needs to be ruled-out in future research (cf., Deroy and Spence, 2013). Another promising result concerns the correlational findings between the magnitude of Stroop effects and the participants' rating of the statement “I am experiencing color when thinking about certain letters” in the study by Colizoli et al. (2012). However, the finding needs to be interpreted with caution as Stroop interference indicates that an association occurs automatically but allows no conclusions about the perceptual nature of the association. Moreover, the statement “I am experiencing color when I see certain letters” did not correlate with Stroop interference. Future studies should also include items such as “I do not experience colors when thinking about or seeing certain letters, but I automatically associate them with colors” to prevent suggestive questioning.

Interestingly, these studies which reported that synaesthetic *experiences* may be, at least in part, acquired later in life are also those which included the largest numbers of inducer-concurrent pairings. Howells (1944) presented his participants with ~30,000 trials of which 95% corresponded to the to-be-learnt associations. The participants in the study of Colizoli et al. (2012) read on average over 100,000 words containing consistently colored letters (i.e., no incongruent letter-color pairs). The success of these training studies follows logically from the fact that every training procedure needs to act against lifelong normal experiences. None of the other studies included more than 10,000 training trials. Moreover, it seems that explicit training procedures (Howells, 1944) lead to stronger effects over shorter time periods than implicit training procedures (Colizoli et al., 2012). Nevertheless, it seems that also implicit training procedures are fruitful to result in potential perceptual effects (Colizoli et al., 2012; Kusnir and Thut, 2012). However, future studies will need to rule out the alternative explanations in the previous paragraph before firm conclusions can be drawn.

The ratio between to-be-learned and not-to-be-learned associations seems to be another crucial factor of the training procedure (cf., Table 1). Generally, there is broad agreement between the different studies that even a relatively short period of training is sufficient to mimic certain aspects (but not the *experience*) of synaesthesia such as Stroop interference for congruent and incongruent inducer stimuli (e.g., Meier and Rothen, 2009; Brang et al., 2011; Rothen et al., 2011). The only exception to this is a study in which all potential combinations of inducers and concurrents were presented equally often. Consequently, the ratio was to the disadvantage of the to-be-learned associations (Cohen Kadosh et al., 2005).

Despite the promising results, even very extensive training durations do not necessarily guarantee to result in phenomenological synaesthetic *experiences*. Probably the strongest overlearned digit-color associations, reported for a non-synaesthetic control, evolved over an extensive period of 8 years through the use of cross-stitch patterns (Elias et al., 2003). In a synaesthetic consistency test the trained control was 100% consistent and the synaesthete was 98% consistent. The control and the synaesthete were also tested using fMRI. Naming colored single-digit numbers revealed a behavioral Stroop effect and similar brain activations for congruently and incongruently colored numbers in the control and the synaesthete. As the control was not interviewed about her subjective phenomenological experiences for numbers, it is open to debate to what extent these resembled those of the synaesthete. However, brain activation in the control and the synaesthete differed in two other tasks. These tasks consisted of simple arithmetic problems, which were either created from dice patterns or presented auditorily, and had to be solved silently. It is open to debate whether this was due to different strategies in solving the specific tasks or due to different phenomenological experiences related to the colors associated with the numbers presented during the tasks.

“Training” during childhood seems more promising and can result, but does by no means guarantee to result, in phenomenological synaesthetic experiences. There is evidence from grapheme-color synaesthesia that repeated exposure to colored childhood toys (e.g., letter refrigerator magnets) early in life leads to synaesthetic *experiences* (Witthoft and Winawer, 2006, 2013) as the following statement indicates: “this insistence on the perceptual nature of synesthesia in at least some cases [including two of those reported here (with synaesthesia acquired from letter refrigerator magnets)] has been invaluable in demonstrating that the color associated with a grapheme can have a great deal more specific content than just associating a letter with a color name” (Witthoft and Winawer, 2013, p. 6). In contrast, a case of monozygotic twins, who both acquired digit-color associations very early in life from a number jigsaw puzzle, does not fulfill our conservative criteria for acquired synaesthesia. When tested at the age of 12, they exhibited a behavioral Stroop effect in a color naming task with colored-digits. However, “they do not report photisms or any sense of perceiving a color, they simply know that zero is pink.” (Hancock, 2006, p. 149). These cases suggest that the development of phenomenological synaesthetic experiences may be more dependent on internal contingencies. In contrast, the nature of the exact association may be more dependent on external contingencies. For instance, training may only influence the exact pairings (i.e., which color is paired to which letter) and may only be effective given certain internal circumstances (e.g., genetic predisposition).

There are other potential candidates for internal contingencies besides a genetic predisposition. As already mentioned in the introduction, synaesthesia is associated with a specific profile of enhanced memory performance (Yaro and Ward, 2007; Rothen and Meier, 2010a; Radvansky et al., 2011; Rothen et al., 2012; Meier and Rothen, 2013b), increased creativity (Rich et al., 2005; Ward et al., 2008; Rothen and Meier, 2010b), and increased self-rated imagery (e.g., Barnett and Newell, 2008; Meier and Rothen, 2013a). Each of these factors alone or in combination may contribute to the emergence of synaesthesia. For example, if in a particular situation, a person is more likely to assign colors to letters, enhanced imagery may result in vivid color experiences and good memory will eventually give rise to the subsequent retrieval of these vivid color experiences during letter processing. This is also in line with the notion that child synaesthetes become more consistent over time or lose the condition entirely (Simner et al., 2009; Simner and Bain, 2013). Moreover, it may be the reason for why no single genetic factor has been identified to cause synaesthesia. The genetic factors to determine the phenotype of synaesthesia may be those which determine the upper limits of creativity, imagery, and memory.

Hence, future training studies may want to account for factors such as creativity, imagery, and memory to further our understanding about the development of synaesthesia. Similarly, it may be worth investigating potential transfer effects of aspects which can be mimicked in training studies. For instance, consistent and automatic associations between graphemes and colors enrich the semantic network and hence, may provide for a richer world of experiences which, in turn, might be one of the features at the core of the benefits of synaesthesia (Meier, in press). Moreover, as some associations may be more easily acquired than others, future training studies should attempt to train other forms than grapheme-color synaesthesia (cf., Deroy and Spence, 2013). Even if it may not be possible at all to acquire synaesthetic experiences, the relevant studies are still informative for cognitive science. For instance, training studies, in combination with neuroimaging and psychophysiological techniques, proved useful to gain insights about phenomenological differences reported by developmental synaesthetes and trained controls. In ERP (Brang et al., 2011; Niccolai et al., 2012) and fMRI (Nunn et al., 2002) studies, synaesthetic experiences were associated with early sensory processes/brain regions in contrast to trained associations in non-synaesthetic controls. Similarly, SCR studies proved useful to contrast synaesthetic *experiences* and semantic associations (Meier and Rothen, 2009; Rothen et al., 2013). Related to this, future studies could aim at increasing the effect of the training by providing bio-feedback.

Before we conclude, we would like to point out that, by considering the very same empirical evidence, a more negativistic view could be adopted which has led others to conclude that “synaesthesia training” and similar terms are not justified (Deroy and Spence, 2013). However, we regard exactly this term as most appropriate because it reflects the ultimate goal of the studies in question to train *specific* inducers-concurrent pairings with perceptual qualities on a subjective phenomenological basis.

## Conclusion

The message for synaesthesia researchers and the interested general public is that there is no solid evidence that synaesthesia can be acquired by training. However, this does not exclude the possibility that synaesthesia can be learned by the appropriate training. Future research is necessary to develop more efficient training procedures. Nevertheless, previous research has clearly shown that some typical aspects of synaesthesia can be learned easily. Future studies will also need to address whether synaesthesia training may lead to transfer effects, such as enhanced memory performance, more vivid imagery, or more creative ideas.

## Author note

Nicolas Rothen is supported by the Swiss National Science Foundation (Grant PA00P1_145370/1).

### Conflict of interest statement

The authors declare that the research was conducted in the absence of any commercial or financial relationships that could be construed as a potential conflict of interest.
